# The effects of COVID-19 lockdown measures on health and healthcare services in Uganda

**DOI:** 10.1371/journal.pgph.0001494

**Published:** 2023-01-23

**Authors:** David Musoke, Sarah Nalinya, Grace Biyinzika Lubega, Kevin Deane, Elizabeth Ekirapa-Kiracho, David McCoy

**Affiliations:** 1 Department of Disease Control and Environmental Health, School of Public Health, College of Health Sciences, Makerere University, Kampala, Uganda; 2 The Open University, Milton Keynes, United Kingdom; 3 Department of Health Policy, Planning and Management, School of Public Health, College of Health Sciences, Makerere University, Kampala, Uganda; 4 International Institute for Global Health, United Nations University, Kuala Lumpur, Malaysia; Burnet Institute, AUSTRALIA

## Abstract

Many countries across the world instituted lockdowns as a measure to prevent the spread of COVID-19. However, these lockdowns had consequences on health systems. This study explored effects of the COVID-19 lockdown measures on health and healthcare services in Uganda. The qualitative study employed focus group discussions (FGDs), household interviews, and key informant interviews (KIIs) in both an urban (Kampala district) and rural (Wakiso district) setting in central Uganda. Fourteen FGDs were conducted among community members, local leaders, community health workers, and health practitioners. Interviews were conducted among 40 households, while 31 KIIs were held among various stakeholders including policy makers, non-governmental organisations, and the private sector. Data was analysed by thematic analysis with the support of NVivo 2020 (QSR International). Findings from the study are presented under four themes: maternal and reproductive health; child health; chronic disease services; and mental health. Maternal and reproductive health services were negatively affected by the lockdown measures which resulted in reduced utilisation of antenatal, postnatal and family planning services. These effects were mainly due to travel restrictions including curfew, and fear of contracting COVID-19. The effects on child health included reduced utilisation of services which was a result of difficulties faced in accessing health facilities because of the travel restrictions. Patients with chronic conditions could not access health facilities for their routine visits particularly due to suspension of public transport. Depression, stress and anxiety were common due to social isolation from relatives and friends, loss of jobs, and fear of law enforcement personnel. There was also increased anxiety among health workers due to fear of contracting COVID-19. The COVID-19 lockdown measures negatively affected health, and reduced access to maternal, reproductive and child health services. Future interventions in pandemic response should ensure that their effects on health and access to health services are minimised.

## Introduction

As COVID-19 progressively spread around the world at the beginning of 2020, many countries scrambled to control the spread of the virus by implementing a range of interventions [1]. These included standard infection prevention and control initiatives such as hand washing with soap and use of alcohol-based hand sanitizers. Later, the wearing of face masks was added to the measures [2]. However, as the pandemic worsened, other more stringent measures to enable social distancing were implemented. These included lockdown measures consisting of curfews, travel restrictions, closure of various institutions, prohibition of public gatherings, and closure of borders [2].

Uganda commenced its response to COVID-19 in early March 2020 after the WHO declared COVID-19 a public health emergency of international concern even before the first case of the disease was confirmed in the country. Several measures were therefore instituted to prevent the spread of COVID-19 in the country. These measures included a national lockdown consisting of night curfew from 19:00hrs to 05:30hrs, suspension of various forms of public transport, restricted movement of people, closure of schools and other institutions of learning, suspension of all forms of public gatherings, and closure of national borders [2]. Several other countries within sub Saharan Africa and beyond also instituted lockdown measures to reduce the spread of COVID-19 [3]. By May 2020 when the first phase of the lockdown was gradually eased, Uganda had a daily average of less than 45 reported new COVID-19 cases. Although the country thereafter briefly experienced a surge in cases, the strict second lockdown that commenced in June 2020 contributed to reduction in reported cases [4]. Uganda arguably minimised and contained community transmission of COVID-19 through the strategy of early implementation of the COVID-19 control measures including the lockdown in 2020 and 2021 [5].

Lockdown measures have short and long-term effects on health and health service delivery in many countries, with children and women most affected [6]. For example, in one modelling study, it was estimated that the impact of COVID-19 and its control measures could increase maternal mortality by up to 38.6%, and a 44.7% increase in child mortality across 118 low- and middle-income countries (LMICs) primarily through the indirect effects of the disease [5]. Recent studies that investigated the effects of COVID-19 on maternal health in Uganda found that several indicators worsened, with an increase in pregnancy complications, as well as maternal mortality and low birth weight, likely due to limited access to care [7, 8]. In addition, child health and well-being were anticipated to be negatively affected by the various lockdown measures in Uganda [9]. Indeed, available evidence indicates an increase in physical, sexual and emotional violence against children [5, 9, 10], child labour [10, 11], as well as food insecurity and malnutrition [10, 11]. There is generally limited empirical data documenting the effects of COVID-19 lockdown particularly in LMICs, hence more evidence is needed to concretize the effects of lockdown measures on the health system. Such evidence will not only be useful in the control of future waves of COVID-19 but also other pandemics. This study therefore explored the effects of COVID-19 lockdown measures on health and healthcare services in Uganda. Our findings provide important insights for various stakeholders on the scope of the health challenges experienced during the COVID-19 lockdowns in both rural and urban settings, in addition to informing the development of interventions to mitigate the negative effects of lockdown measures in the future.

## Methods

### Study area and setting

The study was carried out in both an urban and rural setting. The urban setting was Rubaga Division, Kampala District, while the rural one was Kasanje Town Council, Wakiso District. Kampala and Wakiso districts were selected as the 2 study sites because they had registered the highest cases of COVID-19 in Uganda during the first (November to December 2020) and second (April to June 2021) waves. Rubaga Division in Kampala has 13 parishes which are predominantly urban informal settlements. The division has a projected population of approximately 427,300 people (53.8% female and 46.2% male) [12]. The major economic activities in the area include small scale business such as general retail shops, furniture shops, restaurants and auto-mobile repairs. Kasanje Town Council in Wakiso has seven parishes which are predominantly rural and a few peri-urban. Kasanje Town Council has a projected population of 46,042 people (49.9% female and 50.1% male) [13]. The main economic activities in the area include subsistence agriculture, tourism, small-scale trade and brick laying. The town council has one public health facility (Kasanje Health Centre III), two government-aided (private not-for-profit) health facilities, nine registered (private) clinics, and 22 registered (private) drug shops [13]. Rubaga Division has two government health facilities (Kitebi Health Centre III and Kawaala Health Centre III), 11 private-not for profit facilities, and several private facilities [14]. The health facilities in both study sites provide both preventive and curative health services such as maternal and child health, as well as services for communicable and non-communicable diseases. Prior to the pandemic, inhabitants of the study area had routine access to health services in both public and private sector health facilities. In addition, services for management of childhood illnesses (malaria, pneumonia and diarrhoea) could be obtained from community health workers.

### Study design and participants

The study was qualitative and employed three data collection methods: focus group discussions (FGDs), household interviews, and key informant interviews (KIIs). The different methods were used to get a diverse range of views from participants, so as to allow for triangulation. FGD, household survey and KII guides were used to explore and obtain insights from different stakeholders about the impacts of the COVID-19 prevention measures including lockdown on health and healthcare services in Uganda. The tools included questions on: COVID-19 in Uganda; the lockdown measures implemented by the government; perceptions on the consequences on these measures; effects of the measures on the health system including delivery of services; effects of the measures on women and children; and effects of the measures on household members as well as the broader community. A total of 14 FGDs were conducted, 7 in the urban setting in Kampala district and 7 in rural Wakiso district. The FGDs comprised of 8 to 16 participants, and were categorized into female youth, male youth, adult females, adult males, local community leaders, community health workers (CHWs), and health practitioners. The youth involved in the FGDs were between 18 and 30 years. Categorisation of the FGD participants by age and sex ensured a gendered space for exploration to enhance free expression during the discussions.

The study also used household interviews to obtain their experiences and perspectives on the impact of the COVID-19 prevention measures. A total of 40 household interviews, 20 from each site (urban and rural), were conducted using an interview guide. One interview was conducted per household, and households that participated in the study were identified by community mobilisers to represent high, middle and low-income households. Socio-economic status was assessed based on various household factors including type of employment, location of residence, and house structure. The mobilisers, who had lived in the area for over 20 years, were knowledgeable about the community hence able to identity households that belonged to each of the 3 categories. Before commencement of data collection, the research assistants did an assessment (including observation and asking some questions) to ensure that the households fit within the selected category. In the urban area, 6 high-income, 7 middle-income and 7 low-income households were involved, while 5 high-income, 7 middle-income and 8 low-income households participated from the rural setting. Household heads or their spouses in the respective households took part in the study. These study participants were predominantly female due to their availability at home at the time of data collection. KIIs were conducted to obtain the perspectives of other stakeholders such as policy makers, implementers, international agencies, non-governmental organisations, researchers and the private sector on the impacts of COVID-19 prevention measures. A total of 31 KIIs were conducted, and key informants were purposively selected due to their involvement in the response to COVID-19 control at national and sub-national levels. A summary of the study participants is provided in Table 1.

**Table 1:** Participants involved in the study.

Kampala focus group discussions	Category	Number
	Health practitioners	16
	Community health workers	13
	Community leaders	11
	Female youth	12
	Female adults	15
	Male youth	12
	Male adults	15
	**Total**	**94**
**Wakiso focus group discussions**		
	Health practitioners	8
	Community health workers	12
	Community leaders	12
	Female youth	13
	Female adults	13
	Male youth	12
	Male adults	11
	**Total**	**81**
**Kampala household survey**		
	Low-income	7
	Middle income	7
	High income	6
	**Total (17 female, 3 male)**	**20**
**Wakiso household survey**		
	Low-income	8
	Middle income	7
	High income	5
	**Total (15 female, 5 male)**	**20**
**Key informant interviews**		
	Government ministries	4
	International agencies	7
	Non-governmental organisations	5
	Local government officials	6
	Hospital staff	4
	Professional bodies	1
	Research institutions	4
	**Total (13 female, 18 male)**	**31**

### Data collection

The seven zones from Rubaga Division in Kampala which participated in the study were: Nakulabye, Lubya, Mapeera, Lugala, Lusaze, Namungoona and Kasubi IV. These zones were purposively selected as they were largely comprised of urban informal settlements, with a high population and overcrowded households which increased their vulnerability to COVID-19. In addition, these zones consisted of people with varying socio-economic status which offered varied experiences and perspectives about the impacts of the COVID-19 preventive measures. Participants of the health practitioners’ FGD in Kampala were from Kawaala Health Centre IV which is the largest public health facility in the division and a designated COVID-19 sample collection and case-management facility.

Participants of the community FGDs in Kasanje Town Council, Wakiso District were predominantly from rural zones. The ten zones which participated in the study were: Kasanje, Sokolo, Bweyogerere, Buyege, Jjungo, Koba, Bukalaza, Kikalala, Taba and Sakabusolo. Community FGD participants were selected by the community mobilisers. All the community FGDs were conducted at public places within the respective communities particularly schools and offices of local council chairpersons. Participants of the health practitioners’ FGD in Wakiso were from Kasanje Health Centre III which was also purposively selected as it was a COVID-19 testing site in the area. Participants of the health practitioner FGDs were purposively selected by management of the respective health facility from various departments including paediatrics, laboratory, maternity. The FGDs for health practitioners were conducted at the respective health facilities. The FGDs involved participants creating a timeline of various COVID-19 prevention measures from March 2020 to July 2021. The participants of each FGD then made presentations of how these measures impacted health. The FGDs were audio recorded, with all community FGDs conducted in *Luganda*, the most widely used local language in both study sites. The health practitioner FGDs were conducted in English, and all FGDs were facilitated by 2 female research assistants with prior experience in qualitative research. The research assistants were not known to study participants which facilitated open and objective interaction during data collection.

The participants of the household interviews in both study sites were from the same zones as those who participated in the FGDs. Key informants were mainly selected purposively based on their involvement, expertise and influence regarding response to COVID-19 and its impact on health. Consultation was done by the research team among key stakeholders in the COVID-19 response including from the Ministry of Health and local authorities to ensure appropriate key informants were selected. After they were identified, the research assistants made an appointment with each key informant at a time of their convenience. All tools were developed in English and later translated to *Luganda* (except for the KII guide which was used in English) before they were piloted in a zone in Wakiso district that was not involved in the study. All key informant interviews were conducted virtually via Zoom due to COVID-19 travel restrictions at the time.

### Data management and analysis

The research team that participated in data management and analysis (SN, GBL, DMu) all have expertise and experience in qualitative research. The audio recordings of the FGDs and household interviews were transcribed verbatim and proof-read by a research assistant to ensure that they were accurate. Since the FGDs were mostly conducted in *Luganda*, the transcripts were translated into English, and the translation verified by another researcher. Both the research assistant who transcribed and translated the transcripts, and the researcher who reviewed and verified the translated transcripts (SN) are proficient in both English and the local language, and experienced in qualitative data analysis. The audio recordings of the KIIs were auto transcribed by a software since they were conducted in English. All generated transcripts were then proof-read by another research assistant and edits made to ensure accuracy. The transcripts were then imported into NVivo 2020 (QSR International) software where data analysis was done. Thematic analysis was used to guide the analysis process using the inductive approach [15]. Two researchers read through the transcripts several times to familiarise themselves with the data. Thereafter, the researchers developed codes from the transcripts which were then discussed by the entire team and subsequently refined and revised. The codes were then defined, and several quotes representing different codes were highlighted to develop a code book. The code book was reviewed by the entire research team and modifications made as agreed. Codes that were linked or those that covered a similar subject were grouped to form sub-themes. Related sub-themes were then grouped to develop themes. The final themes obtained from the analysis are the major findings presented in this paper.

### Ethical considerations

The study obtained two stage ethical approval, first from Makerere University School of Public Health Research and Ethics Committee (# 923), and then the Uganda National Council for Science and Technology (# SS881ES). Written informed consent was obtained from all participants, and participation was voluntary. Anonymity was ensured as the participants’ identifying information was not audio recorded during data collection, neither were their names taken. Data was only accessed by the research team to maintain confidentiality and not used for any other purpose. The FGDs were held in accordance with the COVID-19 prevention guidelines in the country. Indeed, both the research assistants and participants were provided with face masks, hand sanitization was carried out frequently, and social distancing was observed. In addition, the household interviews were carried out in open spaces which ensured social distancing as a preventive measure for COVID-19. The FGDs and household interviews were also conducted during a low community COVID-19 transmission period between the first and second waves of the pandemic.

## Results

A total of 175 FGD participants were involved in the 14 FGDs, while there were 40 household survey participants and 31 key informants that also took part in the study. Findings from the study on the impact of COVID-19 lockdown measures of health and health care services are presented under four themes: maternal and reproductive health; child health; chronic disease services; and mental health.

### Maternal and reproductive health

Study participants reported that maternal and reproductive health services were negatively affected by the COVID-19 lockdown measures. These participants revealed that pregnant women and new mothers faced several challenges regarding their scheduled antenatal and postnatal visits. The hindrances included: bureaucracies involved in obtaining travel permits from the authorities; difficulty in accessing transport initially because of the suspension of public transport and curfews, and secondly due to the increased transport fares when public transport was allowed to operate at half capacity. These challenges related to travel affected both urban and rural settings, and were worse especially during the first phase of the lockdown. The need to obtain travel permits and other challenges in seeking healthcare particularly during the first phase of the lockdown resulted in many pregnant mothers delivering either at home, with local traditional birth attendants, or on the way to health facilities.

“*And then when it came to maternal health*, *there was the issue of transport hence many mothers stayed at home and missed or postponed their maternal-related appointments*. *During this period*, *postnatal care including review of mothers and health worker support was lost in the lockdown*.”

Key informant 11, World Health Organization Uganda staff

Health facility related factors such as fear of contracting COVID-19 or being forced to test for COVID-19 while attending antenatal or postnatal visits were reported by study participants. Respondents in urban areas were particularly affected by the temporary suspension of routine maternal health services (including antenatal and postnatal care) in some COVID-19 designated health facilities. Indeed, the government decision to transform some health facilities into strictly COVID-19 treatment sites meant that patients seeking other healthcare services had to look for alternative facilities.

“*I stopped [attending antenatal visits] at 3 months*. *I never wanted to sacrifice myself to COVID-19 because it was easy to catch it from the health facility*. *The benefits of antenatal care seemed less than the risk of dying from COVID-19*.”

Participant 1, low-income household, Kampala

Participants of the health practitioner FGDs and some key informants agreed that provision and access to antenatal and postnatal services was greatly affected by the COVID-19 lockdown. This was attributed to the reduced health workforce as a result of the high cost of transport during lockdown, and the fear of contracting COVID-19 from clients. This created a very high workload for the few health workers available at the facilities in both the urban and rural areas. In addition, more priority was given to COVID-19 rather than maternal health services at the time especially during the early waves of the pandemic. Rural health facilities were more affected by the shortage of health workers due to their remote locations, coupled with the absence of transportation means to take them to work.

“*There were many health workers who could not afford the extra money [due to increased transport during lockdown] and they therefore would not come [to the health facility]*. *This meant that the few who managed to report to duty would bear the burden of the rest of their colleagues who had stayed at home*. *A single health worker would be the midwife*, *laboratory technician*, *clinician*, *dispenser and in case of an emergency that required referral*, *that same health worker would be the transporter because they could not allow a patient to die*.”

Participant 2, health practitioner FGD, Wakiso

Community FGDs and household interviews revealed that access and utilisation of family planning services also declined during the lockdown. This was due to change in family dynamics including the presence of men at home most of the time, and reduced priority for family planning services. Indeed, many families considered getting through the pandemic as their main concern, with less attention given to child spacing. Men being present in some homes meant that some women could not go for family planning services without their partner knowing as was the case before lockdown.

“*During the lockdown*, *husbands used to stop their wives from using family planning*. *Women told us that although some of their husbands did not approve of family planning*, *they would get a chance to access those services earlier when their husbands were away at work*. *But during the lockdown*, *husbands used to be at home all the time*, *and the women could not get a chance to sneak out to get the family planning services they desired*.”

Participant 8, community health worker FGD, Wakiso

Participants of the health practitioner FGDs said that reduced access to family planning services (even when the services existed at health facilities) led to a rise in the number of unwanted pregnancies during the lockdown. In addition, participants of the FGDs and household survey, particularly those in rural areas, added that pregnancies and unsafe abortions among children under 18 years increased during the lockdown. This finding was attributed to children being out of school for a long time during the pandemic.

“*There has been a lot of adolescent pregnancies due to the fact that schools are closed*. *Children are all at home hence there is a lot of interaction among those from different age groups and backgrounds so they end up for engaging in behaviours that result into pregnancy*.”

Key informant 14, Wakiso District Health Team member

Furthermore, participants of the health practitioner FGDs in both the urban and rural settings reported that the adolescent sexual health education clinics at health facilities were suspended for a while during the lockdown. This suspension, which was primarily due to priority being given to COVID-19, limited the routine special support given to this group regarding sexual and reproductive health.

“*Then for adolescent health services*, *I should say that because of COVID-19*, *especially at the beginning of the lockdown*, *there were no outreaches or adolescent health clinics*; *it was just not a priority*. *Therefore*, *many of the adolescent and sexual health services were not taking place*.”

Participant 7, health worker FGD, Wakiso

### Child health

Findings from the FGDs and household survey showed that child health was generally negatively affected by the lockdown measures as a result of the challenges that parents and guardians faced. Participants reported that many children, especially those from low-income households, lacked sufficient and nutritious food especially during the early months of the lockdown. The key informants agreed with this finding and added that it was because many parents survived on income earned on a day-to-day basis hence had little or no savings during lockdown. Indeed, it was reported that lockdown gave such parents no opportunity to prepare for how they would take care of their families. Children from urban households where most of the food was normally bought from markets were more affected than those in rural areas where food was predominantly grown at subsistence level.

“*Another complaint that I got from communities is the high level of malnutrition during the lockdown*. *This was due to the fact that some of these homesteads could hardly access adequate foodstuffs to feed their children because they didn’t have an income or savings at the time*.”

Key informant 14, Wakiso District Health Team member

The key informants as well as participants of the health practitioners and community health worker (CHW) FGDs reported that fewer routine paediatric services were provided due to the COVID-19 lockdown. For example, CHWs reported that they could not carry out their usual treatment of malaria, pneumonia and diarrhoea among children under 5 years due to various reasons such as lack of medicines, and fear of moving around in the community. Health practitioners in both urban and rural settings also reported that services such as immunisation and other paediatric activities were negatively affected by the lockdown. This was largely attributed to health systems and non-health related challenges such as difficulty in obtaining transport to health facilities, increased health practitioner workload, and lack of health facility supplies.

“*As a mother*, *I was affected in that I could not take my child for immunisation as I could not drive my personal vehicle*, *neither could I walk all the way from home to the health facility*. *Therefore*, *I had to postpone the immunisation schedule of my child*.”

Participant 5, health practitioner FGD, Kampala

Participants of the community FGDs and household survey revealed that many community members resorted to self-medication including using local herbs and other local remedies to treat their children when they became ill. These practices reportedly reduced access to health services during the lockdown period.

“*My children became sick and I gave them local herbs because there was no way I could take them to the health facility*. *In addition*, *I would pound and boil lemons and garlic and give my children in addition to the local herbs*.”

Participant 3, women FGD, Kampala

### Chronic disease services

The FGDs and household survey revealed that patients with chronic conditions such as HIV/AIDS, tuberculosis and non-communicable diseases, who needed regular care and medicines, faced numerous challenges during the lockdown. Overall, the condition of patients with chronic conditions worsened, while others died primarily because they could not access the healthcare services that they needed. For instance, due to the abrupt onset of the lockdown, many HIV/AIDS patients were stuck in areas far from where they were registered to receive their medication hence they could not access medicine refills. The suspension of public transport also made it difficult for patients to access health facilities in both the urban and rural settings hence many missed their appointments.

“*Sometimes*, *drugs would delay for the people who needed them the most especially HIV/AIDS and TB patients*. *The patients had to get drugs monthly but the transport means for getting the drugs from the health facilities was a big challenge*. *The same applies to diabetic patients some of whom died because they could not access their medicines*.”

Participant 6, community leaders FGD, Kampala

Both community and health practitioner FGDs reported that there were medicine stock-outs for chronic conditions such as HIV/AIDS, diabetes and hypertension in the public health facilities. The major reason for these stock-outs included the reduced priority accorded to these conditions, as well as disruption in the routine supply chain system due to the pandemic. Rural health facilities which were harder to reach were more affected by stock-outs than urban ones.

“*We noted that at some point last year*, *there were some hospitals that recorded drug and supplies stock outs for patients with chronic health conditions such as diabetes and hypertension*. *The commodities for chronic conditions ran out because of delays at different levels in the supply chain*.”

KII 26, Researcher

In addition, participants of the FGDs and household survey, especially those in urban areas, reported that chronic conditions worsened due to insufficient health education about them during the lockdown. This was because most of the health education messages especially through radio, television and CHWs mainly focused on COVID-19. Other factors such as lack of exercise were cited as risk factors which increased the incidence of NCDs during the lockdown.

“*The lockdown increased the occurrence of many other diseases especially non-communicable diseases such as hypertension*. *This is because people were forced to stay home and did little exercise*. *People were afraid to take walks in the community and hence gained weight which contributed to hypertension*.”

Participant 10, low-income household, Kampala

### Mental health

The study participants revealed that the lockdown led to social isolation from relatives and friends. This situation meant that many mental health support networks were disrupted, thus increasing depression, stress and anxiety within the community. These community networks were predominantly informal including family, friends and religious groups. For example, some participants said that suspension of religious gatherings took away their opportunity to connect with friends and reduce stress through routine communal prayer. Children’s mental health in both the urban and rural areas was also greatly affected during the lockdown due to social isolation from peers leading to depression.

“*When churches were closed*, *access to spiritual health and some forms of psychosocial support provided by religious leaders was cut off*. *In addition*, *many people go to church to meet their friends and socialise hence reducing stress which was not possible during lockdown*.”

Key informant 31, Ministry of Health

In addition, loss of jobs and family income due to the lockdown increased stress among community members, especially those in urban areas who had informal jobs. This result increased depression among the community during the pandemic.

“*People have lost their jobs and they have a lot of stress*. *People’s businesses have been closed yet they have loans to pay*. *Our psychiatric units also reported high cases of depression*. *People are having mental breakdowns due to COVID-19 and the lockdown*.”

Key informant 30, Wakiso District Health Team member

Participants of the community FGDs reported that fear of law enforcement, especially related to curfews, increased anxiety in the community in both the urban and rural settings. Community members were always worried that they would be beaten by law enforcers if they did not observe the curfew protocol or were found participating in communal gatherings. This concern greatly affected their mental health.

“*People were constantly living in fear*. *People could be sitting in a small group of 3 and when they saw Local Defence Units coming closer*, *they became so tense as they were scared of being beaten*. *The fear of security personnel caused a lot of stress in the community*.”

Participant 4, youth women FGD, Wakiso

Participants of the health practitioner FGDs reported that the fear of contracting COVID-19 from the patients that they treated increased stress and anxiety among health workers. The health practitioners also said that the health facilities would sometimes run out of personal protective equipment, making them even more vulnerable to contracting the virus. Participants of the health practitioner FGDs in both the urban and rural settings added that they were stigmatised by the community because people perceived them to have COVID-19 hence they were avoided.

“*During the lockdown*, *I regretted why I was a health worker because there seemed to be a lot of stigma from the community*. *People looked at us as if we are the most infected group with COVID-19*. *In a neighbourhood where people know that you are a health worker*, *they start avoiding you*.”

Participant 5, health practitioner FGD, Kampala

## Discussion

This study sought to describe the effects and impact of COVID-19 lockdown measures on health and health services in rural and urban Ugandan communities with the aim of providing evidence to guide future decision making during pandemics. Participants highlighted that the health of the population including access to various services were negatively affected by the COVID-19 lockdown measures. Participants reported facing difficulties in accessing maternal and reproductive health services such as antenatal care, postnatal care and family planning mainly due to transport challenges. Child health was also reported to have been affected by increased child malnutrition, reduced access to services and mental challenges. In addition, routine paediatric services such as immunisation and treatment of childhood illnesses declined during the lockdown. Participants also revealed that the prevention and management of chronic conditions such as HIV/AIDS and NCDs, as well as mental health was negatively affected by the implementation of the lockdown across the country. These effects, observed in both the urban and rural settings, were mainly due to measures such as suspension of both public and private transport, and night-time curfew that greatly reduced access to health services. The study findings emphasize that whereas lockdown measures may be needed to control disease spread during a pandemic, negative consequences affecting health may emerge [3]. These findings highlight the importance of careful consideration of lockdown measures to maximize benefit and minimise harm to the population during the management of pandemics.

Maternal health was negatively affected due to implementation of COVID-19 lockdown measures where access to antenatal and postnatal services declined. These findings concur with those from a study conducted in three East African countries where midwives reported that suspension of transport during the lockdown posed challenges for pregnant women accessing essential maternal health services [16] and may have increased pregnancy-related complications and maternal mortality. The suspension of both private and public transport in Uganda greatly hindered access to essential maternal health services including health facility deliveries, yet no alternative measures were instituted to ensure continuity of care. A study conducted in four sub-Saharan African countries showed that Kenya instituted measures such as multi-month dispensing of antenatal care related supplements and medications as well as telephone antenatal care sessions [17], strategies that were not considered in Uganda. Although the Ugandan government promised that pregnant women would get access to ambulances to transport them to health facilities if they contacted their local leaders, anecdotal evidence suggests that such ambulances were not readily available, forcing many women to resort to traditional birth attendants [16]. The decline of postnatal services during the COVID-19 lockdown was also affected by reduction in home visiting carried out by CHWs. These findings emphasize the importance of planning for the continuity of various maternal health related services during future responses to pandemics.

Our study revealed that access to family planning services declined due to lockdown measures which in turn led to an influx of unplanned pregnancies during the pandemic in both urban and rural areas. Indeed, there was reduced prioritization of family planning services during the lockdown periods in the country. In addition to transport challenges in accessing health facilities for family planning services, outreach activities including those supported by CHWs were reduced [16]. Such reduction in services during lockdown greatly affected access to health services including for family planning. As an example, adequately trained and equipped CHWs are proven to be a viable option especially in areas which do not have an established tele-medicine structure [18] yet their work was greatly affected by the lockdown. The extended closure of schools during lockdown also contributed to the increase in teenage pregnancies especially in rural areas. The extended stay at home exposed youth to risky sexual behaviours including cross-generational relationships [10]. Yet during the lockdown, there was absence of suitable support systems that could have raised their awareness about the dangers of engaging in sex and how to deal with such sexual encounters. Strategies to keep children and youth engaged during lockdowns to minimize their engagement in sexual activities should be planned for in the future management of pandemics with extended school closure.

The findings of our study showed a decline in child health such as an increase in malnutrition among children in both the urban and rural settings. Malnutrition among children in LMICs was projected in studies conducted during the early stages of the pandemic [19–23]. Indeed, our findings are similar to those from earlier studies conducted both in Uganda [6, 23–25] and other LMICs [26–28] which found increased levels of malnutrition among children. Malnutrition in children may have lowered their immunity and made them more susceptible to other diseases. Our study also revealed that routine paediatric services such as immunisation and treatment of childhood illnesses were negatively affected. Immunisation services offered at both health facilities and in the community were significantly slowed during the lockdown [17]. It is believed that low rates of immunisation across the country during the pandemic may have resulted in the recent polio outbreak in Uganda [29, 30]. Due to transport challenges in accessing health services during the lockdown, home treatment of childhood infections using herbs were common. The use of herbs in management of diseases has been reported in other studies conducted in Uganda [31]. Although lockdown measures were aimed at curbing the spread of COVID-19, future interventions should be implemented in ways that minimize severe and long-term impact on children’s health and wellbeing.

The lockdown measures were reported to have devastating effects on the health of those with chronic conditions such as HIV/AIDS, tuberculosis and NCDs. The increased challenges in accessing drugs among patients were highlighted in our study. Regarding access to HIV/AIDS drugs, our findings differ from those obtained from a study conducted in South-Western Uganda which found sustained access to health services among patients [32]. This difference could have been due to the presence of a community HIV/AIDS programme which ensured that HIV/AIDS drugs were delivered to the patients. However, the study [32] found increased stigma among HIV/AIDS patients which could have been due to the pandemic including the fear of dying from COVID-19 hence potentially leading to poor mental health outcomes. This finding therefore calls for enhancing attention to mental health services among HIV/AIDS patients and other vulnerable groups during pandemics. For other chronic conditions such as NCDs, other studies conducted in Uganda also established that lockdown measures disrupted the supply-chain of medicines and reduced health-seeking behaviours [33–35]. People with chronic conditions consistently need their medicines to remain in optimal health particularly during such pandemics as they are a vulnerable group. Equitable access to drugs and other health services among such patients should therefore be streamlined before, during and after implementation of lockdown measures.

Poor mental health outcomes due to social isolation, loss of income, and fear of contracting COVID-19 in both urban and rural settings emerged as a major finding in our study. This could be attributed to the abrupt and strict implementation of the lockdown measures which left people with little time to mentally and financially prepare for the extended periods of staying at home. The novelty of COVID-19, reports of high morbidity and mortality rates globally, and information overload from media may have contributed to the anxiety and fear of contracting the disease. Similar findings were documented in studies from other LMICs which showed increased prevalence of depression and anxiety during the pandemic [36–38]. The lockdown measures instituted in Uganda also worsened the already weak mental health infrastructure in the country. Therefore, strategies to support mental health during future lockdown periods such as improving family and social support systems are vital for general wellbeing.

Our study had some strengths and limitations. The triangulation of data sources and methods was a strength of our study. Indeed, our data was obtained using three different methods (KIIs, FGDs and household survey) and from a diverse range of participants at national, sub-national and community levels. This provided a comprehensive range of varied insights and perspectives on the effects of the COVID-19 lockdown. Furthermore, our study collected data from both urban and rural settings hence providing demographically varied experiences of the lockdown. A limitation of our study is that only 2 districts in the central region were involved hence some findings may not be generalisable to other areas in the country. Purposively sampling districts with high levels of COVID-19 could have also over-estimated the health impacts due to the lockdown. In addition, KIIs were conducted virtually due to the COVID-19 restrictions which may have limited the advantages of face-to-face interviews such as observing and probing on non-verbal cues.

## Conclusion

The COVID-19 lockdown measures negatively affected health and reduced access to maternal, reproductive and child health services. In addition, continuity of health services for patients with chronic conditions reduced. Future lockdowns aimed at minimising disease spread during pandemic response should ensure that their effects on health and access to health services are minimised. In addition, interventions should be accompanied by strategies to monitor unexpected consequences and propose mitigation measures. Policy makers and other stakeholders should also build the adaptive capacity of the health system to ensure it remains resilient during pandemics.

## Supporting information

S1 Data (DOCX)
