# Supplementary material for: The effects of COVID-19 lockdown measures on health and healthcare services in Uganda
Source: PLOS Glob Public Health. 2023 Jan 23;3(1):e0001494. doi: 10.1371/journal.pgph.0001494 (PMC10021763; doi:10.1371/journal.pgph.0001494)
Supplement: S1 Data — (DOCX) [file pgph.0001494.s001.docx]

**The effects of COVID-19 lockdown measures on health and healthcare services in Uganda**

| **Theme** | **Sub-themes** | **Meaning unit** |
| --- | --- | --- |
| Maternal and reproductive health | Antenatal and postnatal care | “I stopped at 3 months. I never wanted to sacrifice myself to COVID 19, I was always saying that we can die of other diseases but not COVID 19. But God helped me and I was able to give birth to my baby very well.” (Low Income HH, KII 1, Kampala)  “Then postnatal like immunization services, I may say because of COVID for the beginning when we were in lockdown, there were no outreaches. So that means all postnatal services, including immunization, were not taking place. Then later when we resumed, the services are also not as it used to be, because this is not like before when mothers would be at the facility and you give the health education talk. So, some of those opportunities are being missed because of those COVID-19 restrictions, like social gatherings being restricted.” (Health Educator, Local Government, KII 30, Wakiso)  “…in attempting to contain the disease, there was a strict lockdown and it significantly affected utilization of health services especially RMNCH…even for childhood diseases, immunization lagged behind, many children failed and that time we were quite worried that we will lose the gains in immunization and in fact, lost days of immunization increased significantly during that time. So those were the two main things which we thought, utilization of childhood services especially the preventive health services and the maternal services, they were grossly impacted.” (Doctor, Practitioners Association, , KII 21, Kampala)  “The clinics like the antenatal clinic, post-natal clinic were all suspended and later reopened on a different campus. Because even when we first opened people were afraid to pass near the gate or attend these clinics because they knew there were COVID cases there. But with sensitization of course, through political leaders, local stations, people got to know that the other clinics were not very close to the COVID isolation unit. But now the clinics are up and running.” (Nurse, ERRH, KII 31, Wakiso)  “Then postnatal like immunization services, I may say because of COVID for the beginning when we were in lockdown, there were no outreaches. So that means all postnatal services, including immunization, were not taking place. Then later when we resumed, the services are also not as it used to be, because this is not like before when mothers would be at the facility and you give the health education talk. So, some of those opportunities are being missed because of those COVID-19 restrictions, like social gatherings being restricted.” (Health Educator, Local Government, KII 30, Wakiso)  “The pregnant women also knew that when they get pregnant, they should go to the health facilities for check-up and delivery and medical attention but now, all that was put aside and they all focused on COVID 19.” (Youth Men, FGD, Participant 1, Kampala)  “…some of the services were stripped and scaled back for some time, like immunization and antenatal.” (HW, CHW, Participant 8, Kampala)  “…in attempting to contain the disease, there was a strict lockdown and it significantly affected utilization of health services especially RMNCH…even for childhood diseases, immunization lagged behind, many children failed and that time we were quite worried that we will lose the gains in immunization and in fact, lost days of immunization increased significantly during that time. So those were the two main things which we thought, utilization of childhood services especially the preventive health services and the maternal services, they were grossly impacted.” (Doctor, Practitioners Association, KII 21, Kampala)  “The pregnant women also knew that when they get pregnant, they should go to the health facilities for check-up and delivery and medical attention but now, all that was put aside and they all focused on COVID 19.” (Youth Men, FGD, Participant 1, Kampala)  “We found lower-level facilities where we’d have two wards female and male. One of those would be turned into a COVID-19 ward, so that the maternity ward or the delivery ward, if they had any room, had to be shared by many other patients. And you know what that means. But also, it was never a priority. I had a chance to visit one of the referral hospitals in eastern Uganda and talked to the in charge, who openly told me that for maternal and child health care services and specifically, pregnancy unless it is an emergency, we cannot open it up. We have no health workers to attend to them. I have no one to attend an emergency at theatre, so I cannot say they are working optimally but I understood her case” (Implementor, UN, KII 24, Kampala)  “The pregnant women I think they were affected in a bad way, now someone who had just come to swallow drugs [from attending antenatal services] and they can’t walk home, they get a boda-boda in pain and they are beaten up and some of them ended up giving birth on the way.” (High Income, KII 1, Kampala) |
|  | Birth and delivery | “Then also, there was a lot of increase in maternal deaths as a result of long distances and failure to reach hospitals. There were a lot of pregnancy related complications due to reduced services, they could not seek any guidance and hence developing or detecting the effects at a later time, sometimes it would be too late.” (HW, FGD, Participant 7, Wakiso)  “So, we saw the increased the number of maternal deaths during the lockdown because people would not access the health facilities in time. We also saw a number of neonatal deaths increase in that initial lockdown because there was that delay in accessing health facilities for care. Yeah.” (Doctor, NGO, KII 2, Kampala)  “I gave birth to my child at night, I didn’t go back to the health facility to get medicines because it was very far and yet the private health facilities were there but you wouldn’t be having money to go there to be treated so, it was really hard for some of us.” (Low Income HH, KI 1, Kampala)  “Mothers would come and say they delivered from home not because she liked, but because she was dodging to go to the hospital because she doesn't have enough money So what I have realized is I was receiving so many still births plus mothers saying they were using TBAs … It [still birth] could be attributed to poor antenatal care maybe to taking herbs because if one is not going for antenatal care and take traditional herbs called ‘mumbwa’ thinking it is good for their babies but those herbs could lead to still births. ” (Midwife, Private Sector, KII 28, Wakiso)  “I remember the case of a pregnant woman who they brought because they were trying to seek for permission from RDC to come to the hospital, but it was too late, by the time they arrived at the hospital she had passed away.” (Implementor, NGO, KII 5, Kampala)  “…we were so much affected especially these ladies that were going to give birth, The RDC was the only person authorized to write a letter to allow these pregnant mothers to go to the health facility. They were so much affected in a way that someone urgently in need of health services like treatment, drugs and others could not get them without that letter. They later realized that there was only one RDC and the people were so many and living in very distant places and could not all come from their places to the RDC.” (Men, FGD, Participant 9, Wakiso) |
|  | Family planning | “During the outbreak, it was not too much because our husbands used to stop their wives from using family planning. Because some ladies would tell you that she used family planning but the husband does not agree with it. But now when the man goes for work, the lady gets a chance to sneak out for the injection and then she goes back. But during the lock down, the husbands used to be at home, and the women could not get time to sneak out to get the injection.” (CHW, FGD, Participant 8, Wakiso)  “In terms of health seeking behaviour from our end we observed a marked increase in demand for our services, family planning, post-natal care and maternity services, women and girls are at home, girls are out of school, boys are out of school and have a lot of time and a lot of sexual activities going on. We observed and increased in demand for health services on the free side. So, we had more clients coming for the free services, we particularly observed an increase in the number of young people in their teens coming for contraception and especially in our unpaid services that are largely rural based. To be specific, the proportion of young people coming to our outreaches for family planning has gone up from the previous 13% to now at 17%. That's really a huge increase in terms of actual clients who walk through our outreaches.” (Country Director, NGO, KII 29, Kampala)  “It was bad, I went to the health centre once my back was hurting and they told me I needed to bring my ID because I was on family planning, so they sent me to get my ID which I had to pick from a far village where I had left it, the ID shows the family planning I was on, so I just left it because I had no means of going to pick it. They told me in order for them to remove the family planning I had to show that ID but I could not.” (Middle Income HH, KII 6, Wakiso) |
|  | Adolescent pregnancy | “We are continuing to register 840 abortions every week in this country. That is quite a very big number for one to tell you that every week we are registering this number of abortions. They are being done and some of them illegally causing these kids to lose their lives, while others are being done and they do not to lose their lives, but they are illegal. There are illegal abortions that are being carried out.”(Implementor, UN, KII 27, Kampala)  “…of course, the impact of the covid-19 according to the interactions that I have had during this time of COVID, you realize that there's been a lot of teenage pregnancies due to the fact that schools are closed. So, pupils, children and students are all at home there’s a lot of mix up and they learn lots of things when they mix up with these different cultures, different age groups, so they end up for behaving that way and resulting into pregnancy.” (Health Inspector, Local Government, KII 14, Wakiso)  “Some of the bad habits I can say is among the girl children, whereby they felt like they were grown-ups so they would engage in sexual relationships as well as marriage as they were uncontrollable. You would find that the young boy that was convincing the girl didn’t always have enough time for her because she would be home for 2 weeks and then go back to school. They have now been home for almost a year so it is really hard to control that child.” (Youth Men, FGD, Participant 1, Kampala)  “Some are really very unfortunate and unforgettable. I'll never forget some time, I think November last year, there was this lady, I didn't meet her myself, but a workmate did. She met her and she was just she said she doesn't know what she did to this world to deserve what she has been going through. So, she has four daughters, who were all pregnant. Yes, four daughters and pregnant from two boys from the neighbour. Now just look at that four daughters pregnant and from young boys in the neighbourhood. So, she didn't know what to do, whose children would they be. Will they take care of them? So that's just one of those extreme scenarios, but there are very many of that kind.” (Doctor, WHO, KII 11, Kampala)  “I am telling you, a young girl at 8 years is able to get pregnant and when they see their fellow young men of 14 years and children these days are mad depending on what they watch on movies, on their smart phones so all the things that are filled on those phones, you find children seated and you get shocked at what they are watching. I tend to be close with them and during that, I find them watching things and I see them and I get shocked too.”(Low Income HH, KII 6, Wakiso) |
| Child health | Infant malnutrition | “Another thing that I got from communities, there's been a high level of malnutrition due to the fact that some of these homesteads can hardly access adequate foodstuffs to manage their children because they don’t have a variety of foodstuffs.” (Health Inspector, Local Government, KII 14, Wakiso)  “Some of them had left home without food, some mothers would say that my child is sick and I do not have food, so connecting to our offices where we work from was also a problem. You would find that these people who were supposed to help them were not in offices and these were poor people staying in the slums of Bwaise and Nakulabye, most of the children became malnourished.” (HW, FGD, Participant 1, Kampala)  “Some HIV/AIDS infected children were so much affected in that, there was little food, there was little to drink, so the conditions were not nice at all especially for the children” (Community Leaders, FGD, Participant 7, Kampala) |
|  | Routine paediatric services (immunisation, VHT child health services) | “Yes, they increased the prices and they were too high. My child once got typhoid and when I took her to the health facility, they asked me for $ 29 USD yet we were in COVID so there was no where I could get that money from.” (Low Income HH, KI 1, Kampala)  “Then this other part was for me as a mother, I was affected in that I couldn’t take my child for immunization, I could not drive my personal vehicle and still, I couldn’t walk all the way from home to the health facility so I had to postpone the immunization schedule.” (HW, FGD, Participant 5, Kampala)  “And then when it comes to child health, there is the first vaccine that is given at the hospital so they miss out if the child is delivered at home and now post-natal care has immunization services, review of the mother so all that cascade of support is lost in a lockdown situation.”(Doctor, WHO, KII 11, Kampala)  “The children have been affected the most because where a mother would be able to go on a boda boda [motor cycle] or a taxi with a child to a facility now they have to walk and if there’s anybody that had had to walk with a five year old, when they get tired, you have to carry them and you have to think of refreshments and you have to think of toilets. It was very burdensome on families, especially those with small children under the age of 10.” (Implementor, NGO, KII 22, Kampala) |
|  | Child mental health | “Firstly, it has a psychological effect child are so much affected. So, a child can become depressed and can end up getting depression. and, you know, it's a disease, a sickness, because you see your mother being tortured and that depresses you. Secondly, it makes children to develop hatred against themselves, and also it perpetuates violence, when a child is exposed to violence, he/she develops hatred and so that hatred causes them to also become violent by themselves, so it perpetuates violence amongst the children and among the people. But also, that violence at home makes some of them to go ahead and commit suicide because they cannot stay in that home and they become suicidal.” (Implementor, NGO, KII 5, Kampala |
| Chronic care services (NCDs, HIV, TB) | Medicines and health workers unavailable/inaccessible | “Sometimes, drugs would delay for the people who needed them the most especially the HIV/AIDS patients, the person had to get drugs monthly but the transport means for getting the drugs from the health facility where they were was a big problem. Same applies to the diabetic people, they died in large numbers, because they could not access the medicines. All these diseases killed more people than COVID because there were no means of transport to the health facilities for treatment.” (Community Leaders, FGD, Participant 6, Kampala)  “And then also the general population, people had to go for their routine drug refills, like those patients on TB, HIV medication but somehow, somewhere they were not able to access their drugs yet you know like ARVS, a patient is not supposed to miss even a day. But somehow some of those people had to miss their refills because they were not able to move.” (Health Educator, Local Government, KII 30, Wakiso)  “But for chronic conditions like hypertension, diabetes mellitus it is the biggest challenge since the responsibility on when to return has always been with the patient, the clinician clearly recommends in their notes the review date but they don't do active follow up like we have been doing for HIV and TB. The clients are expected to take note of the date and report back. So that is where we see significant loss to follow up.” (Doctor, Local Government, KII 17, Kampala)  “…but we know and are aware that people are not accessing testing the way they should have done so, but we are also aware that we are seeing an increase in the number of people indulging in non-marital sexual acts. The increase in that category is high. But also, the most worrying part is that those people are not utilizing condoms.” (Implementor, UN, KII 27, Kampala)  “Yesterday I was listening to NBS news, I think the head of cancer institute said they used to receive around 100 patients in a day, but because of this lockdown, they are now receiving around 20 patients. And it is the same with the heart institute in Mulago. The number of patients seeking medical attention for heart problems that I'm talking about heart problems because even them they are affected, people who no longer come and the personnel are being diverted to COVID-19 management.” (Implementor, NGO, KII 5, Kampala)  “…the lockdown came in when I already had drugs for 2 months but it eventually got finished. You know when you are hypertensive and you are a farmer. You do not feel it very well. But I cannot leave the village and come to town, I feel headache, neck pains and a very intense heartbeat but when I have my hoe and I am digging, I don’t feel it but if at all I feel it, I get some herbs and put in warm water and I drink it and I eventually get fine and may be also taking that garlic.” (Low Income HH, KII 5, Kampala)  “I have a neighbour who at one time I had to share my medicine with because we were taking the same medicine, the son who used to care was affected by COVID, he used to work in Nakasero and she did not have the money to buy the medicine, then I told her let me give you half of mine, we shared then when I got some money I topped up…” (High Income HH, KII 1, Wakiso)  “When you look at other comorbidities like HIV, people who are living with HIV have been most hit, they can't get refills, those who would get refills would share with neighbours.” (Implementor, UN, KII 9, Kampala) |
|  | Missed appointments | “I would like to add on what others have said that there was rampant spread of the other disease among people because all focus was put on COVID 19, even the health workers focused on COVID 19 and so the other patients with other diseases like HIV, cancer, many had those deadly diseases and they were not being cared for as they used to be before COVID.” (Youth Women, FGD, Participant 1, Kampala) |
| Mental health | Fear and anxiety (for the community due to measures and COVID-19) | “The worst was when one of us got infected with COVID, as my colleague said, and I personally tried to reach our supervisors to get means of helping this person, the results were out and this person was positive and then our supervisors were like let her stay at home, contact this person at Mulago and you know the Mulago team was like we are only dealing with VIPs so it seemed like no one was there for us and that tortured me psychologically.” (HW, FGD, Participant 5, Kampala)  “…they would tell us that we are going to send vehicles to come for you, still that would make you emotionally unstable, because, you are going not because you want to work much but because of the responsibility and the pressure, as well as the regulations so mentally, emotionally, it was not something easy much as we were not on site to face the patients but wherever we were, emotionally affected by what was going on at that facility where we were supposed to be.” (HW, FGD, Participant 11, Wakiso)  “On the mental health effect, me as a health worker, to be honest at that time I regretted why I was a health worker because there seem to be a lot of stigma from the community. People pointed at us as if we are the most at risk and in a neighbourhood where people know that you are a health worker, people started avoiding us.” (HW, FGD, Participant 5, Kampala)  “The burden on our side as health workers was huge. It was tiring. Our emotional state, we were scared. In a day you would lose over 20 patients in just a couple of minutes or hours. And it was no longer predictable, someone could come in walking and the next day they would be dead. It was that scary. So physically, emotionally it was so draining for the health workers…”(Nurse, ERRH, KII 31, Wakiso)  “….when you look at the regional facilities government had gazetted as a treatment centre, you find that most of the health issues were put aside, so you might find that, for example, the mental health area has been turned into an isolation centre, meaning that the people who have issues with mental health aren't going to be able to access the services they need. So, you find that the attention has been shifted from the normal usual issues that affect the population. But now the attention is put on COVID even when you come with the usual malaria and other things you find doctors have been prepared and are there for COVID. You are not given attention. Some areas have been taken away and have been sealed off for COVID. So, the health sector has also been affected.”  (Policy marker, GoU, KII 20, Kampala)  “Living on pressure, people would be sitting in a small group of 3 and then see LDUs coming closer, a hypertensive person would just down, so people were scared, they got stressed and were asking themselves on how they will survive. Let’s say there is no food at home, someone would be worried about how they will survive, where will I get the drugs, this person is sick, so that caused pressure.” (Youth Women, FGD, Participant 4, Wakiso) |
|  | Stigma (health workers / victims; NCDs, HIV and TB) | “Very many patients of HIV get their medication from far from their homes. The reason being that they don't want people around them to know. Now you're telling them, that to get their next dose you will be assisted, they will not ask you for anything. So that then sent many in hiding. They said over my dead body, unless a friend picks it up for me and then we meet somewhere, and I get it and go back home. So, stigma now became apparent, reality that we had not bothered to attend to… Some of them had to even disclose to family members forcefully because they needed help, you know, when you need help, you have to speak up. So, they were forced to disclose. And you know what comes with the forced disclosure, why you had been scared, also now comes up. And the other indirect consequences around that.” (Implementor, UN, KII 24, Kampala)  “But you know issues with things like HIV, things like STIs young people will not move to the nearest facility because they are known there, their parents and their families are known there. And if a young person goes and tells another in the neighbourhood that she's pregnant, the chances are high that the nurse or counsellor will tell her family.” (Implementor, UN, KII 8, Kampala)  “Of course, there is stigmatization within the community, more so on some of the chronic diseases. That people know, because when you go somewhere they are easily identified. The first question for the patients especially for HIV, they don't want people to know that they are HIV. That means you are not going to have that patient come. Yet we also have stigmatization among TB patients, sicklers so it affects the entire health service delivery and quality of service so it is complex.” (Biostatistician, ERRH, KII 6, Wakiso):  “Last year when we went into lockdown there as a group of key populations that was arrested. Simply because they live together, and people did not think it was moral for people of the same sex living together. So, these young people were arrested and I think spent close to a month in prison with a lot of embarrassment, but we know that there is no law that prohibits Uganda of whichever sex from living together. The law might be on other issues, but living together, we could live together as girls in the same house, but for people to moralize some of these things, I think it was uncalled for and these people were locked down. Many of them had ARVs and during the lockup, they were not allowed to take their ARVs for almost a month, you can imagine someone who is positive and they've gotten off treatment what it would be.”(Implementor, UN, KII 9, Kampala) |
|  | Social isolation | “So, in cases where churches are closed, that means access to spiritual health and some forms of psychosocial support provided by religious leaders was tampered with. And of course, you know that mainly women listen to religious leaders. And on the broader aspect of health and public health, spiritual health is really paramount.” (Doctor, MoH, KII 31, Kampala)  “…we are in Uganda and Africa at large; everyone believes that there is something that helps them to survive. There are some people whereby when they get sick, they go to pastors so that they can pray for him or go to a traditional healer but all these were impossible as the places of worship were closed as well as the shrines. Those that were open were so far away so it was hard and many people suffered in that way.” (Men, FGD, Participant 3, Kampala)  “But I mean, but I also know families that have had COVID, it is not easy to isolate at home, so again that has a psychological effect on children. There are few homes you may find they can afford to cut off a bedroom, then you can’t mingle with these guys, children don't want to know that they can’t get closer to you. So, imagine those have been affected psychologically, being at home but the dad says don't come close to me, or the child but you can’t because you're trying to protect them, so they may not understand why you're doing it or appreciate that but I think socially also psychologically it could have been impactful to them as well.” (Researcher, University, KII 7, Kampala) |
|  | Stress (job security/loss of income) | “Our mental health was affected, you had been used to staying at home with your children when the husband has gone to work and will return late at night, but he is now at home, he is looking out for the mistakes that you do at home, you have done this, you have spoiled the soap and left the soap in the water, it was hard for us and we hated ourselves and you would look for where to go and there is nowhere to go because there are no means of transport and the man who you would not expect to be coming back at 9pm, would stay at home for the whole day.” (Low Income HH, KI 1, Kampala)  “Now the old people, after losing their jobs, the landlord is demanding you as a tenant, you have the responsibility of your children, so that stress disorganized them and caused people to get so many diseases, be it hypertension, be it diabetes and these are caused by stresses.” (Community leaders, CHW, Participant 4, Kampala)  “People have lost their jobs, they have stress they have nothing to eat, maybe because people are together, they are discovering that maybe this wife has been cheating on me, maybe because of finding out maybe the phone messages, all the issues are happening. Another thing that has happened that may not be particular to women, but cuts across, those mental health challenges because of too much stress. As I told you, loans, people’s businesses have closed up. Our psychiatric units also reported high cases, of depression. People are mental breakdowns, not because that they use it to have them, but because of the psychological torture that they are having. You can imagine you've just got a loan of five million and these people especially in the informal sector, don't go to banks, so these are people are supposed to pay on a daily basis. So, in that process people are having all sorts of stress and in the process, the mental cases in the district are also going on an increase.” (Health Educator, Local Government, KII 30, Wakiso)  “…those that could not handle the situation died of stress because I have evidence of some people who died due to that, the bank demands them, everything demands him and that would lead to death.” (Youth Men, FGD, Participant 10, Kampala)  “These people, some of them even murdered each other on seeing that they have a lot of responsibilities that they cannot fulfil… one man, he realized that they had a lot of children and he couldn’t manage the responsibility and he thought that killing his wife would be the solution.” (Low Income HH, KII 1, Kampala)  “…no, it was worrying thoughts because I was not making money. For someone who was independent to be in a position where you cannot afford something. Someone can call me right now and tell me my child is sick but I don’t have even any money right now.” (Middle Income HH, KII 6, Wakiso) |
